# The mechanisms that regulate *Vibrio parahaemolyticus* virulence gene expression differ between pathotypes

**DOI:** 10.1099/mgen.0.000182

**Published:** 2018-05-29

**Authors:** Nicholas Petronella, Jennifer Ronholm

**Affiliations:** ^1^​ Biostatistics and Modelling Division, Bureau of Food Surveillance and Science Integration, Food Directorate, Health Canada, Ottawa, ON, Canada; ^2^​ Department of Animal Science, McGill University, Ste-Anne-de-Bellevue, QC, Canada; ^3^​ Department of Food Science and Agricultural Chemistry, McGill University, Ste-Anne-de-Bellevue, QC, Canada

**Keywords:** *Vibrio parahaemolyticus*, Gene, Regulation, TDH, TRH, RNAseq

## Abstract

Most *Vibrio parahaemolyticus* isolates found in marine environments are non-pathogenic; however, certain lineages have acquired genomic pathogenicity islands (PAIs) that enable these isolates to cause human illness. The *V. parahaemolyticus* PAI contains one or both of two toxins: thermostable direct haemolysin (TDH) or TDH-related haemolysin (TRH) and type III secretion system 2 (T3SS2). Recently, a few *V. parahaemolyticus* isolates that do not have this PAI were obtained from clinical samples, and there has been interest in determining whether these isolates possess novel virulence factors. In this investigation, we have selected four *V. parahaemolyticus* isolates: a canonical pathogenic strain containing TDH, TRH and T3SS2; two strains from clinical cases which do not contain a PAI; and an environmental isolate which also does not contain a PAI. For each isolate, we analyzed differential gene expression after crude bile exposure. Several enteric bacterial pathogens are known to use bile as a signal to enhance virulence gene expression. We have shown that in the *tdh-*positive *trh-*positive pathotype gene virulence gene expression was not up-regulated in response to crude bile, strongly indicating that the current dogma of virulence gene regulation in *V. parahaemolyticus* needs to be revisited and separately investigated for each pathotype. In addition, we have created a list of genes of interest that were up-regulated in the non-canonical pathotypes which may contribute to virulence in these isolates.

## Data Summary

1. RNA-seq raw sequence-reads for 04-2548 under control conditions have been deposited in the Sequence Read Archive (SRA); accession number: SRR3930257 (url: https://trace.ddbj.nig.ac.jp/DRASearch/run?acc=SRR3930257).

2. RNA-seq raw sequence-reads for 04-2548 after crude bile exposure have been deposited in the Sequence Read Archive (SRA); accession number: SRR3930378 (url: https://trace.ddbj.nig.ac.jp/DRASearch/run?acc= SRR3930378).

3. RNA-seq raw sequence-reads for 09-5357 under control conditions have been deposited in the Sequence Read Archive (SRA); accession number: SRR3930425 (url: https://trace.ddbj.nig.ac.jp/DRASearch/run?acc= SRR3930425).

4. RNA-seq raw sequence-reads for 09-5357 after crude bile exposure have been deposited in the Sequence Read Archive (SRA); accession number: SRR3930426 (url: https://trace.ddbj.nig.ac.jp/DRASearch/run?acc= SRR3930426).

5. RNA-seq raw sequence-reads for ISF-29-03 under control conditions have been deposited in the Sequence Read Archive (SRA); accession number: SRR3930427 (url: https://trace.ddbj.nig.ac.jp/DRASearch/run?acc= SRR3930427).

6. RNA-seq raw sequence-reads for ISF-29-03 after crude bile exposure have been deposited in the Sequence Read Archive (SRA); accession number: SRR3930434 (url: https://trace.ddbj.nig.ac.jp/DRASearch/run?acc= SRR3930434).

7. RNA-seq raw sequence-reads for 04-1290 under control conditions have been deposited in the Sequence Read Archive (SRA); accession number: SRR3930403 (url: https://trace.ddbj.nig.ac.jp/DRASearch/run?acc= SRR3930403).

8. RNA-seq raw sequence-reads for 04-1290 after crude bile exposure have been deposited in the Sequence Read Archive (SRA); accession number: SRR3930424 (url: https://trace.ddbj.nig.ac.jp/DRASearch/run?acc= SRR3930424).

**Impact Statement**

Bile is a major component of the human intestine. It is essential for digestion, controls the population size of the microbiota, and helps to prevent pathogen colonization. However, several enteric bacterial pathogens have bile resistance mechanisms and some use bile as a signal to activate expression of virulence genes to enhance their infective abilities. From previous work it has been established that *Vibrio parahaemolyticus* can resist bile-mediated killing and can use bile as a signal to express genes on its primary pathogenicity island. However, each of the studies conducted to examine virulence gene regulation, in response to bile, in *V. parahaemolyticus* used the same strain. *V. parahemolyticus* is a genetically diverse species and there are at least three different pathotypes, which each contain a slightly different pathogenicity island. There is potentially a fourth *V. parahaemolyticus* pathotype which does not contain a recognizable pathogenicity island. In this study, we have examined gene expression in four different *V. parahaemolyticus* strains in response to crude bile and found that different pathotypes respond to the presence of bile in very different ways, in terms of virulence gene regulation. These findings indicate that researchers performing future studies that are attempting to understand virulence in this species should take into account that different pathotypes appear to have different mechanisms to regulate gene expression from their pathogenicity island. In addition, bile sequestration, which has been proposed as a treatment for *V. parahaemolyticus* infection, may only be effective for one of the three established pathotypes.

## Introduction

*Vibrio parahaemolyticus* is a Gram-reaction-negative, halophilic bacterium that naturally occurs in marine environments, including estuaries. Most *V. parahaemolyticus* strains are non-pathogenic, however, some have acquired virulence factors that can result in human illness when individuals are exposed through the consumption of contaminated seafood [1]. *V. parahaemolyticus* is recognized as the leading cause of food-borne illness associated with the handling and consumption of raw or undercooked seafood [1]. The most common manifestation of a *V. parahaemolyticus* infection is watery diarrohea accompanied by abdominal pain and nausea, however, acute illness can also be more severe with symptoms including a dysentery-like illness or septicaemia [2]. Since most cases of *V. parahaemolyticus* infection are self-limiting, the true rate of infection is probably much higher due to under-reporting.

Non-pathogenic *V. parahaemolyticus* strains outnumber pathogenic strains both in estuary settings as well as in seafood. Pathogenic *V. parahaemolyticus* isolates are differentiated from their non-pathogenic counterparts by the presence of several virulence factors. Pathogenic isolates generally have at least one of two major toxigenic virulence factors, thermostable direct haemolysin (TDH) [3] and TDH-related haemolysin (TRH) [4]. During infection TDH and TRH are involved in cytotoxic and haemolytic activity [5–7]. All *V. parahaemolyticus* isolates contain a type III secretion system (T3SS) commonly called T3SS1, and pathogenic strains typically contain a second T3SS called T3SS2 [8]. T3SS2 occurs on a pathogenicity island (PAI) on chromosome II, alongside TDH and TRH, and encodes the toxin genes (*VopA*, *VopC*, *VopL* and *VopT*) as well as the T3SS2 gene cluster [9, 10]. T3SS2 has been shown to contribute to enterotoxicity in both TDH- and TRH-containing isolates [6, 11]. T3SS2 derives from two separate genetic lineages: T3SS2α is typically found in association with the *tdh* gene, while T3SS2β is found in lineages that contain either the *trh* or both the *trh* and *tdh* genes [12]. Pathogenic and non-pathogenic strains also tend to differ with respect to the presence of two type VI secretion systems (T6SSs). T6SS2 is found in all *V. parahaemolyticus* strains, while T6SS1 is mostly associated with clinical isolates, and may also play a role in virulence [13, 14]. The T6SS is composed of 13 essential genes and a variable number of non-essential genes, including various toxigenic effector proteins [15]. On the basis of the presence or absence of different virulence genes each clinical *V. parahaemolyticus* isolate can be assigned to one of four pathotypes (Table 1) [16].

**Table 1.** Genomic content of each *V. parahaemolyticus* pathotype

	*V. parahaemolyticus* pathotypes			
	TDH-positive	TDH-positive	TDH-negative	TDH-negative
	TRH-negative	TRH-positive	TRH-positive	TRH-negative clinical isolates
Toxins in PAI	TDH	TDH and TRH	TRH	No PAI
T3SS2	T3SS2α	T3SS2β	T3SS2β	No T3SS2
T6SSs	T6SS1 and T6SS2	T6SS1 and T6SS2	T6SS1 and T6SS2	T6SS1* and T6SS2

*T6SS1 is associated with some but not all isolates in this category.

Isolation of *V. parahaemolyticus*, from patients presenting with typical vibriosis symptoms, that lack *tdh*, *trh*, and T3SS2 has been reported in several recent publications [17–22]. This breaks with the canonical dogma surrounding the pathogenesis of members of the genus *Vibrio*, therefore, the ability of *tdh*-negative and*trh*-negative isolates to cause illness is controversial. It is know that seafood often contains multiple *V. parahaemolyticus* strains, and it has been suggested that if infection with multiple strains occurs and at least one strain carries virulence factors, then non-pathogenic strains, not directly involved in the illness, could be isolated from the infected individual – masking the presence of virulence factors in the truly pathogenic strain [19]. Despite this explanation for *tdh-*negative and *trh-*negative clinical isolates, there are also lines of evidence that indicate that some *tdh-*negative–*trh-*negative strains may be able to cause illness. For example, during a coinfection study, three sick patients produced 30 *tdh-*negative and *trh-*negative isolates, and despite multiple attempts no other enteric pathogens or pathogenic *V. parahaemolyticus* could be cultured from these individuals [19]. A comparative genomic analysis of *tdh-*negative and *trh-*negative strains isolated from either clinical or environmental sources identified some key genetic differences between clinical and environmental *tdh*-negative*–trh*-negative isolates, including a novel T6SS effector protein [16]. Therefore, it is still unclear if *tdh*-negative and *trh-*negative clinical isolates are a rare cause of vibriosis and have novel virulence factors that warrant future study, or if these isolates are merely co-infecting alongside pathogenic isolates; and if these isolates are only involved in co-infection, do these *tdh*-negative and *trh-*negative clinical isolates have an increased ability to survive *in vivo* relative to their environmental counterparts?

Bile is a major component of the human intestine. It is essential for digestive processes and is known to up-regulate expression of virulence genes in several enteric pathogens [23–25]. In the human gut, bile is one of the most long-term, potentially bactericidal threats to enteric pathogens. The entire small intestine contains some amount of bile, which ranges from 0.2 to 2 % (w/v) depending on time, day, diet, and individual differences [26]. Bile also probably prevents the overgrowth of commensal bacteria in the small intestine [25]. Several enteric bacterial pathogens have an abundance of genes to neutralize the effects of bile, and also utilize bile as a signal to up-regulate important virulence genes for an efficient infection [25]. This appears to be true for certain strains of *V. parahaemolyticus*. Crude bile has been shown to increase production of the TDH protein [27], increase bacterial adherence to Int-407 cells *in vitro* [28] and up-regulate expression of T3SS2α and TDH at the level of transcription [29, 30]. Bile exposure has also been shown to very effectively mimic the *in vivo* environment for transcriptomic analysis in *V. parahaemolyticus*, since, based on RNA-seq in an experimental procedure, 69 *V. parahaemolyticus* genes were induced in the presence of bile, and 53 of these genes were induced in the intestine of a rabbit model [31]. However, it should be noted that each of the experiments that have evaluated the regulatory response of *V. parahaemolyticus* to bile exposure have used the RIMD2210633 strain, which contains a *tdh* gene but lacks the *trh* gene [29, 31]. In this investigation, we explored differential gene expression in response to crude bile in *V. parahaemolyticus* strains with different genetic traits. We selected a clinical isolate containing both *tdh* and *trh* (04-1290), two clinical isolates without either the *tdh* and *trh* genes (04-2548 and 09-5357) and an environmental isolate that also lacked both toxin genes (ISF-29-03). In the strain containing both toxin genes, we found that the genes associated with TDH, TRH and T3SS2β are not up-regulated in response to bile-salt exposure – indicating that there are critical differences in the regulation of virulence genes between the different pathotypes of *V. parahaemolyticus* that have been overlooked until now. This study also provides a list of which genes are up-regulated in clinical isolates that lack *tdh* and *trh*, in response to crude bile exposure. Understanding the differences between gene expression in clinical and environmental isolates, with the same genetic profile (*tdh*-negative, *trh*-negative and T3SS2-negative), in response to bile, which is known be an important signal factor for gene regulation in several enteric pathogens, has provided valuable information about this controversial pathotype [29].

## Methods

### Strains and culture conditions

Four strains of *V. parahaemolyticus* were used in this study: 04-2548, 09-5357, ISF-29-03 and 04-1290 (Table 2). Each of these strains had been previously sequenced and extensively characterized in earlier studies [16, 21, 32, 33] (Table 2). Stock cultures were removed from storage at −80 °C and streaked onto Tryptic Soy Agar (TSA)-2N and grown overnight at 35 °C. A single well-isolated colony was selected to inoculate 10 ml of Tryptic Soy Broth (TSB)-3N. This inoculum was incubated at 35 °C and followed at OD_600_ until the OD_600_ was approximately 0.6, approximately 4.5 hr. Test cultures were then supplemented with 0.04 % crude bile (CAS 8008-63-7, Sigma Aldrich) as indicated, and further incubated for 30 min. Control cultures were growth under the same conditions, but were not given crude bile during the final 30 min of incubation. Each experiment was carried out in triplicate.

**Table 2.** Isolates used in this study

Strain number	Virulence genes	Isolation source	Genome accession number	Transcriptome SRA accession number (control/bile salt exposure)
04-2548	T6SS1	Clinical	JTGS0	SRR3930257/SRR3930378
09-5357		Clinical	JTGT0	SRR3930425/SRR3930426
ISF-29-03	T6SS1	Environmental	LFYM0	SRR3930427/SRR3930434
04-1290	*tdh*, *trh*, T3SS2β, T6SS1	Clinical	JXVK0	SRR3930403/SRR3930424

### Total RNA isolation

After crude bile gene induction, 500 µl of culture was added to 1 ml RNAprotect Bacteria Reagent (Qiagen). The cell suspension was then transferred to a microcentrifuge tube and incubated for 5 min at room temperature to stabilize the mRNA. Next, the cell suspensions were centrifuged at 8000***g*** for 10 min to pellet the cells, and the supernatant was decanted. The cell suspension was then stored at −80 °C until RNA was purified.

RNA was purified using the RNeasy Mini Kit (Qiagen) according to the manufacturer’s recommended protocol: 'Purification of Total RNA from Animal Cells using Spin Technology' with minor modifications. Bacterial cells were removed from storage at −80 °C and 0.1 ml TE buffer (10 mM Tris.Cl, 1 mM EDTA, pH 8.0) containing 1 mg lysozyme ml^−1^ was added to the cells. The manufacturer’s RLT buffer was added to this solution to further disrupt the cells, and the protocol was followed as described in the manual from this point. Each total RNA sample was double eluted into 30 µl elution buffer and stored at −20 °C until use. If the presence of DNA was detected in the sample by PCR of the 16S rRNA gene using the 27F 5′-AGAGTTTGATCMTGGCTCAG-3′ and 1492R 5′-CGGTTACCTTGTTACGACTT-3′ primers, TURBO DNase (Ambion by Life Technologies) was used to remove it according to the manufacturer’s instructions. The quality and quantity of total DNA free RNA obtained was determined using the Agilent 2100 bioanalyzer and RNA 6000 Nano chips (Agilent). All RNA used in this investigation had a RNA integrity number greater than 8.

### Enrichment of mRNA and sequencing

The rRNA was removed from each sample using the Ribo-Zero Magnetic Kit for Gram-Negative Bacteria (Illumina) as per the manufacturer’s instructions, and the mRNA sample was analyzed with the Agilent 2100 bioanalyzer and RNA 6000 Nano chips to ensure successful rRNA removal. The TruSeq Stranded mRNA Sample Prep Kit (Illumina) was used to prepare the mRNA for sequencing, according to the manufacturer’s instructions, with a few modifications. Briefly, precipitated mRNA from the rRNA removal step was re-suspended in 21.5 µl of Fragment, Prime, Finish Mix, and 19.5 µl of this mix was added to separate wells of a 96-well RNAase-free PCR plate and incubated at 94 °C for 8 min. Double-stranded cDNA was synthesized, 3′ ends were adenylated, adapters were ligated and DNA fragments were enriched using the reagents included in the TruSeq kit (Illumina) according to the manufacturer’s instructions. Each library was quantitated using Quant-iT (Invitrogen) according to the manufacturer’s instructions, and then diluted to a 10 mM concentration in Tris-HCl 10 mM, pH 8.5 with 0.1 % Tween 20 and pooled. Pooled libraries were diluted to a concentration of 4 pM and loaded into a MiSeq Ragent Kit v3 (150-Cycle) (Illumina). All of the samples were sequenced using the Illumina MiSeq M01308 Sequencing platform (Illumina). Each of the RNA-seq experiments was carried out in biological triplicate.

### RNA-seq analysis

Differential gene and transcript expression analysis was essentially carried out as described by Trapnell *et al*. [34], and a detailed description of our pipeline and parameters can be found in the supplementary methods. Briefly, each of the draft-genomes of strains used in this study were downloaded from NCBI and processed by removing contigs of less than 1000 bp to improve sequence-read mapping. RNA-seq sequence reads were then aligned to each of the respective reference genomes using TopHat and Bowtie [35, 36]. Gene annotations were obtained by using PROKKA to directly generate annotations for each reference genome [37]. Cufflinks was used to assemble each of the reads into transcripts [38]. Differential expression analysis, which compared the genes regulated by crude bile induction with the control, was performed by Cuffdiff – part of the Cufflinks package. Differentially expressed genes were identified as a result of averaging triplicate measurements. Figures denoting differential gene expression were generated using CummeRbund. Raw RNA-seq sequence-reads were deposited in the Sequence Read Archive (SRA) under the study accession number SRP078919.

## Results and discussion

### RNA-seq profiling of *Vibrio parahaemolyticus*

To begin to elucidate how various *V. parahaemolyticus* pathotypes (Table 2) respond to crude bile, we performed RNA-Seq analysis on RNA isolated from mid-exponential-phase cultures of four different strains, both exposed to and not exposed to crude bile (all culture experiments were performed in triplicate). Each sample had greater than 1 million sequence-reads associated with it Fig. S1 (available in the online version of this article). Due to targeted depletion of the rRNAs the majority of reads corresponded to annotated open reading frames (ORFs). Based on the findings of Haas *et al*., this level of sequencing depth should be enough to detect a significant number of genes which are differentially expressed by twofold or more [39].

**Fig. 1.**
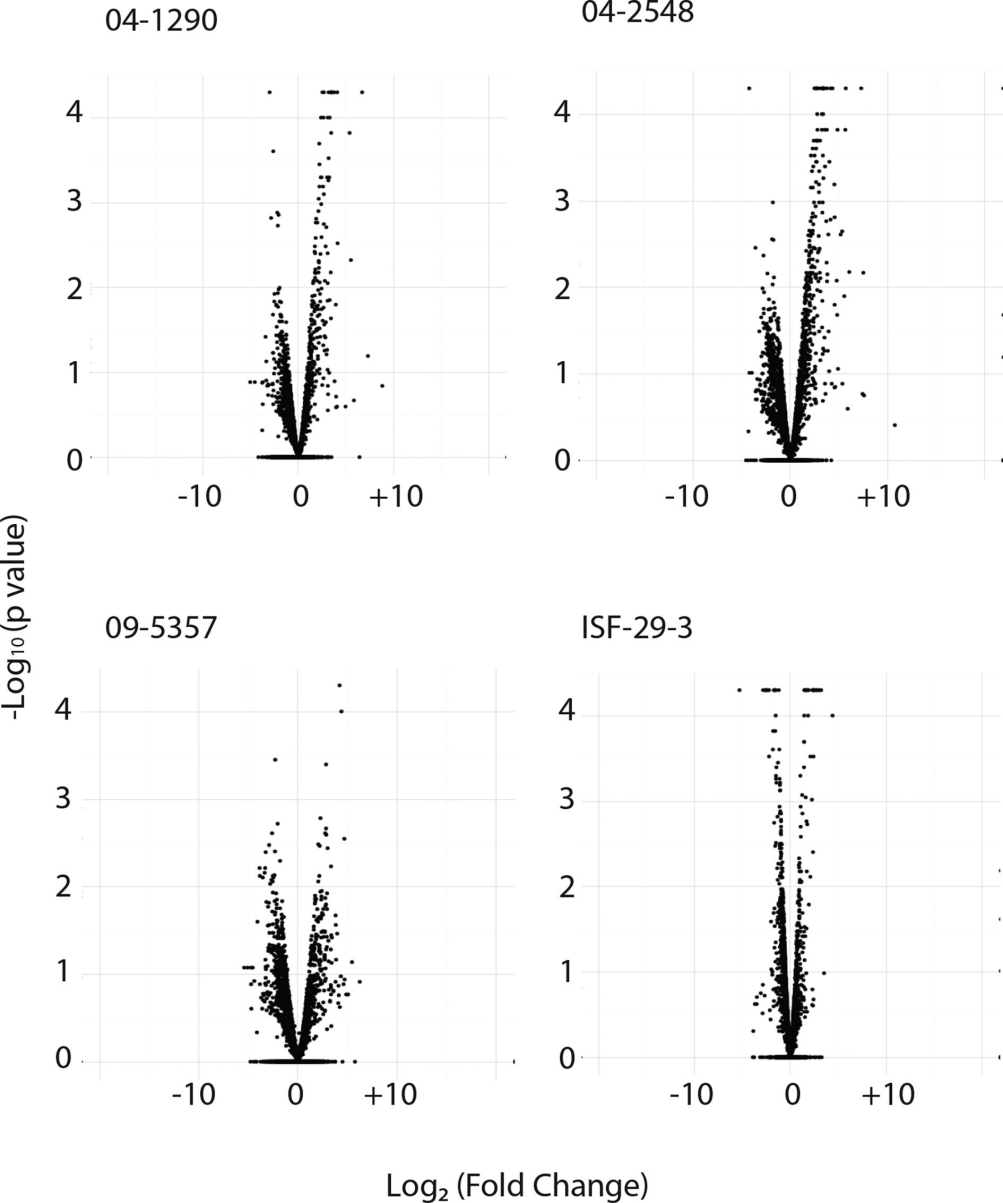
Volcano plots of total genes found to be differentially regulated in each strain.

To identify genes that were differentially expressed in TSB-3N cultures and during crude bile exposure we compared the RNA-seq data for these conditions using CuffDiff, which is a package within the Cufflinks software, that allows for differential expression analysis with RNA-seq data [38]. Genes were considered differentially expressed when both of the following conditions were met: (1) genes showed at least a twofold change after triplicate samples are averaged and (2) the *P* value was less than 0.05 (Fig. 1). When data from the triplicate control cultures and crude bile exposure were averaged, 99 of 4645 annotated ORFs were found to be differentially regulated in response to crude bile in strain 04-1290 (Table S1), 193 differentially regulated genes out of 4995 were identified in 04-2548 (Table S2), 34 differentially regulated genes out of 4843 were identified in ISF-29-03 (Table S3), and 104 of 5124 genes were differentially regulated in 09-5357 (Table S4). It is notable that the fewest differentially regulated genes were observed in the ISF-29-03 strain, which is an environmental isolate that would be very unlikely to encounter crude bile during its life-cycle. The two clinical isolates (04-2548 and 09-5357) that do not contain known virulence genes were more similar, in terms of the number of genes differentially regulated, to the canonical pathogenic *V. parhaemolyticus* isolate (04-1290) than to the environmental *V. parahaemolyticus* (ISF-29-03) isolate.

### Generalized stress proteins

In both the 09-5357 and 04-1290 strains, the stress protein H5 was up-regulated 2-fold. The universal stress protein UspE was also up-regulated 2.5-fold in the 09-5357 isolate. UspE expression has been shown to be up-regulated during growth inhibition caused by the exhaustion of any of a variety of substances or by the presence of toxic agents in *Escherichia coli* [40]. Up-regulation of generalized stress proteins makes sense in the context of exposure to crude bile.

### Multidrug efflux transporters

In each of the four strains of *V. parahaemolyticus* examined in this study, genes associated with multidrug efflux pumps were up-regulated after exposure to crude bile. Crude bile caused a greater than a 2-fold increase in the expression of a multidrug transporter MdtA in each of the four strains examined. In *E. coli* MdtA is known to be up-regulated in the presence of crude bile and conifers resistance to bile [41, 42]. Thus, the up-regulation of MdtA would be expected in this study. Each of the four strains also has a greater than 2-fold increase in the expression of MdtN, which is an unrelated putative multidrug efflux pump membrane fusion protein that, given its simultaneous up-regulation, may also be involved in the bile-salt stress response.

Various other export systems were found to be up-regulated in different strains. In the 09-5357 isolate the genes associated with arsenical resistance, which confer resistance to arsenic via extrusion from cells, as well as a toluene efflux pump outer membrane protein were both found to be up-regulated twofold in response to crude bile exposure. Also in the 09-5357 strain, a four-protein macrolide efflux pump (PROKKA_02394–PROKKA_02397) appeared to be both co-regulated and up-regulated 3.8-fold in response to crude bile exposure. In the 04-2548 strain genes associated with a macrolide efflux pump, a toluene efflux pump and a cation efflux pump were each found to be up-regulated approximately twofold in response to crude bile.

Enteric bacterial pathogens are exposed to bile in the small intestine where bile is abundant, and it is well established that efflux pumps are required for survival in this environment [25]. The importance of efflux pumps to the survival of other pathogenic members of the genus *Vibrio in vivo* is also well established. In *Vibrio cholerae* several efflux pumps have been shown to be required for bile resistance in the intestine including: the TolC system [43, 44], VexAB [45] and VprAB [46]. The VexAB efflux transporter appears to be an important part of the *Vibrio vulnificus* response to bile exposure, although it is not up-regulated during bile exposure [47]. This is an important observation, since it indicates that importance during stress response is not always perfectly correlated with induction of gene expression. In previous work with *V. parahemolyticus* RMID2210633, over-expression of the *vmeTUV* multidrug efflux transporter was associated with increased bile resistance [48], and a different multidrug transporter (VP1713–VP1717) was shown to be up-regulated due to bile exposure [29]. Overall, our data agree with findings for other enteric bacterial pathogens, other species of the genus *Vibrio* and other strains of *V. parahemolyticus*, which indicate that efflux transporters are an important part of the response to bile. However, while each of the clinical isolates appeared to have several efflux transporters that were up-regulated during exposure to crude bile, in the environmental isolate (ISF-29-03) only the *mdtA* and *mdtN* genes were differentially regulated. This observation indicates that the *tdh*-negative–*trh*-negative clinical isolates express genes which would be associated with increased survival in the intestine more than the *tdh*-negative–*trh*-negative environmental isolate.

### Pilus assembly proteins

The pilus assembly proteins CapB and TadB were both up-regulated 2.6-fold in the 04-2548 strain, and in the same strain a co-regulated three-gene locus encoding a fimbrial chaperone protein, an outer membrane usher protein and a putative secreted protein was also up-regulated. In certain pathogenic *E. coli* bile exposure leads to the induction of pilus assembly genes, resulting in increased adherence to intestinal epithelial cells [49]. It is possible that up-regulation of pili assembly genes in the 04-2548 strain results in increased adhesion in this isolate.

### Amino acid synthesis, catabolism and transport

Various genes involved in amino acid metabolism were differentially regulated in each of the four strains investigated in this study. In the environmental isolate (ISF-29-03) an arginine transport protein was up-regulated in response to exposure to crude bile exposure. In 09-5357 arginine deiminase, which is involved in the breakdown of arginine, was down-regulated. In the 04-2548 isolate, arginine N-succinyltransferase, which is also involved in arginine and proline breakdown, was up-regulated. In the 09-5357 isolate, a tyrosine-protein kinase gene *wzc*, the product of which is required for extracellular polysaccharide colanic acid synthesis, was down-regulated. In 04-2548 a glutamine ABC transporter permease, a histidine transport system permease and an amino acid carrier protein were each up-regulated.

A putrescine transport protein was up-regulated 4.6-fold in the 09-5357 isolate and 5.7-fold in 04-2548 isolate. Putrescine is an organic compound that is related to amino acid catabolism and is toxic to cells in large quantities. In *V. cholerae* putrescine down-regulates the expression of the toxin-coregulated pilus (TCP), and TCP is critical for host colonization [50]. Also in the 09-5357 and 04-2548 isolates a glutathionine S-transferase was up-regulated approximately threefold in response to crude bile exposure. Glutathionine S-transferases are involved in binding, transformation and detoxification of a wide variety of toxins in bacteria [51], and in this context it may be involved in the generalized stress response of these particular strains to crude bile. These two potential detoxification mechanisms were up-regulated in the two clinical isolates that lack known virulence factors, but not in the environmental isolate. Having additional detoxification machinery may allow this pathotype to survive better in the host than environmental isolates and therefore result in an infection.

### Fatty acid metabolism

In the environmental isolate (ISF-29-03) a nicotinamide transport system was up-regulated twofold in response to crude bile exposure. In two isolates (09-5357 and 04-1290) two genes: biotin carboxylase, biotin carboxyl carrier protein of acetyl-CoA carboxylase, which were located beside each other in both strains and likely to be co-regulated, were both down-regulated in response to bile. Two homologous genes (VP2880 and VP2881) were observed to be downregulated in response to crude bile exposure in *V. parahaemolyticus* RMID2210633 [29]. Both of these genes are involved in fatty-acid biosynthesis. In the 09-5357 isolate long-chain fatty acid CoA ligase was down-regulated in response to crude bile exposure, but this same gene was up-regulated under the same conditions in 04-2548. In earlier work, fatty acid metabolism has been shown to be one of the systems which was heavily affected in *V. parahemolycicus* in response to crude bile exposure. Genes associated with fatty acid oxidation (VP0029, VP0030, VP2208, VP2209) and fatty acid transport (VP2213) were up-regulated when the cells were exposed to crude bile; while genes associated with fatty acid biosynthesis (VP1591, VP1592) and a different fatty acid transport gene (VP2212) was down-regulated [29]. Very little literature exists surrounding enteric pathogen gene expression in response to crude bile exposure, however, given that differential expression of the genes involved in fatty acid metabolism appears to be common to several strains, this is something that should be investigated in the future.

### Type I secretion systems (T1SS)

Type I secretion is carried out by translocation machinery composed of three proteins that span the cell envelope [52]. In a T1SS one protein forms an outer membrane channel and two are cytoplasmic membrane proteins, an ATP-binding protein and a membrane fusion protein [52]. TolC is an outer membrane channel that can be associated with several different combinations of cytoplasmic membrane proteins to function together as a T1SS. In each of the four strains we investigated, a TolC gene was up-regulated at least twofold in response to crude bile exposure, and in two strains (04-2548 and 09-5357) there was up-regulation of an entire T1SS including MacA, TolC and MacB. In this instance MacA is a periplasmic membrane fusion protein, MacB is an ATPase [53]. Crude bile has been shown to induce the expression of a T1SS in *E. coli* where TolC can be instrumental in intestinal survival via excretion of crude bile [54]. In *E. coli* TolC can be involved in virulence via secretion of enterotoxins [55]. In *V. cholerae* the TolC channel is essential for survival in the intestine, participates in the crude bile stress response and is responsible for secreting the RTX cytotoxin [56]. The observed up-regulation of an entire T1SS in this in response to crude bile in the two clinical isolates without known virulence factors indicates that there could be an unknown toxin being secreted via this system.

### Type III secretion systems

In this investigation only one isolate (04-1290) possessed the *V. parahaemolyticus* pathogenicity island that is associated with virulence in *V. parahaemolyticus* and includes a T3SS, *tdh* and *trh* [16]. Statistically significant differential regulation of this pathogenicity island was not observed. In addition, when the raw dataset was inspected without statistical criteria being applied, we failed to detect any differential expression of this system. At first, this appears to conflict with results reported in previous work, which has clearly demonstrated that the T3SS2α as well as the *tdh* gene are up-regulated in response to crude bile exposure [29, 30]. Up-regulation of the pathogenicity island that contains *tdh* and T3SS2, in response to bile, is controlled by VtrABC. VrtA and VrtC interact to form a protein dimer on the surface of *V. parahaemolyticus* that together bind to crude bile and send a signal to VrtB which then triggers expression of the TDH toxin [30]. However, there is one important distinction between these studies: a different strain of *V. parahaemolyticus* (RIMD2210633) was used in all previous studies and this strain contains the T3SS2α and a *tdh* gene, while the 04-1290 isolate has a T3SS2β and both the *tdh* and *trh* genes [16]. The genome of the 04-1290 strain was searched and it did not contain the VtrABC genes. We conducted a more extensive search of the 49 isolates sequenced in our previous work [16], and determined that only isolates of the *tdh*-positive–*trh*-negative pathotype contained the VtrABC genes, isolates from the other pathotypes did not. Taken together this data indicates that the T3SS2 and PAI from different pathotypes of *V. parahaemolyticus* are not regulated by the same mechanism.

### Potential novel virulence factors

In one of the clinical isolates without known virulence factors (04-2548) a hemolysin was found to be up-regulated approximately threefold during crude bile exposure (Table S2). The gene encoding this hemolysin was 711 bp in length and was not homologous to TDH or TRH. However, when this gene was searched in the NCBI database using BLASTn the closest match (at 100 % homology) was a hemolysin from *V. parahemolyticus* CDC_K4557 [20]. *V. parahemolyticus* CDC_K4557 is another clinical isolate, obtained from the stool of a patient showing symptoms consistent with *V. parahaemolyticus* infection, with no known virulence factors [20]. Finding a gene in more than one clinical *V. parahaemolyticus* isolate without known virulence factors and observing it to be up-regulated in conditions known to mimic infection [30], flags it for further investigation as a potential novel virulence factor.

Two RTX toxins were shown to be up-regulated over threefold in the 04-2548 isolate, including a virulence metalloprotease precursor as well as a leukotoxin in response to crude bile exposure (Table S2). In species of the genus *Vibrio* metalloproteases have been shown to have toxic effects on oyster larvae [57]. Leukotoxins are generally cell-type- and species-specific RTX toxins [58], and are known to be pore-forming toxins in other species of the genus *Vibrio*, including *V. vulnificus* and *V. cholerae* [59]. The cytotoxicity of both of these genes should be evaluated in human cell lines to determine if either or both have roles in virulence.

### Type VI secretion systems

All *V. parahaemolyticus* isolates, including each isolate examined in this study, have a T6SS2. The isolates 04-2548, ISF-29-03 and 04-1290 each also contained a T6SS1 gene locus, which is generally only found in clinical *V. parahaemolyticus* isolates [13]. The 04-2548 isolate has a second alternate T6SS2, not previously observed in *V. parahaemolyticus*, which is 66 % similar, based on nucleotide sequences, to a T6SS2 identified in *V. cholerae* [16]. Several genes within the *V. parahaemolyticus* T6SS2 locus were up-regulated in 04-2548 but down-regulated in both 04-1290 and 09-5357 when exposed to crude bile. Expression of the genes associated with the T6SS1 in each of the isolates, as well as the genes associated with the alternate T6SS2 in the 04-2548 isolate, were not affected by exposure to crude bile.

During an earlier study that looked at regulation of the T6SSs in *V. parahaemolyticus* strain RMID2210633, it was found that T6SS1 was always expressed at below 37 °C with both high and low salt concentrations, and when *V. parahaemolyticus* was grown at low cell density, in low-salt media T6SS2 was expressed [60]. Our experiments, were carried out in high-salt media; however, expression of the T6SS2 was readily observed in three of the four strains and was even up-regulated in 04-2548 in response to the addition of crude bile. These observations indicate that, like T3SS2, the T6SS2 might be regulated via different pathways in different pathotypes of *V. parahaemolyticus*.

### Conclusions

In this investigation, we have characterized the transcriptomic response of four different *V. parahaemolyticus* isolates, a *tdh*-negative *trh*-positive isolate (04-1290), two clinical isolates without *tdh* and *trh* (04-2548 and 09-5357) and an environmental isolate also lacking both *tdh* and *trh* (ISF-29-03) to crude bile exposure. We have shown that the canonical *V. parahaemolyticus* virulence genes, *tdh* and *trh*, as well as the T3SS2 are regulated by different mechanisms in the different pathotypes of *V. parahaemolyticus*. While it is well established that expression of the *tdh* gene and the T3SS2 are up-regulated by bile-mediate activation of the transcription factor VtrB [61], we report here that in members of the *tdh*-positive–*trh*-negative pathotype neither *trh* nor the T3SS2 are up-regulated by bile, the *vtrB* transcription factor is not present in the genome and, therefore, that the *trh* and T3SS2 genes in this pathotype must be regulated by other mechanisms. Overall, the results from this study indicate that there are important differences between pathotypes of *V. parahaemolyticus*, w should be considered when characterizing mechanisms of pathogenesis.

## Data bibliography

1. Ronholm J, Petronella N. Sequence Read Archive SRR3930257 (2016).
2. Ronholm J, Petronella N. Sequence Read Archive SRR3930378 (2016).
3. Ronholm J, Petronella N. Sequence Read Archive SRR3930425 (2016).
4. Ronholm J, Petronella N. Sequence Read Archive SRR3930426 (2016).
5. Ronholm J, Petronella N. Sequence Read Archive SRR3930427 (2016).
6. Ronholm J, Petronella N. Sequence Read Archive SRR3930434 (2016).
7. Ronholm J, Petronella N. Sequence Read Archive SRR3930403 (2016).
8. Ronholm J, Petronella N. Sequence Read Archive SRR3930424 (2016).

## Supplementary Data

44 Supplementary File 1

## Supplementary Data

703 Supplementary File 2
