# Supplementary material for: The mechanisms that regulate Vibrio parahaemolyticus virulence gene expression differ between pathotypes
Source: Microb Genom. 2018 May 29;4(6):e000182. doi: 10.1099/mgen.0.000182 (PMC6096935; doi:10.1099/mgen.0.000182)
Supplement: Supplementary File 2 [file mgen-4-182-s002.pdf]

Fig. (S1)

Click here to download Figure Figure S1.pdf

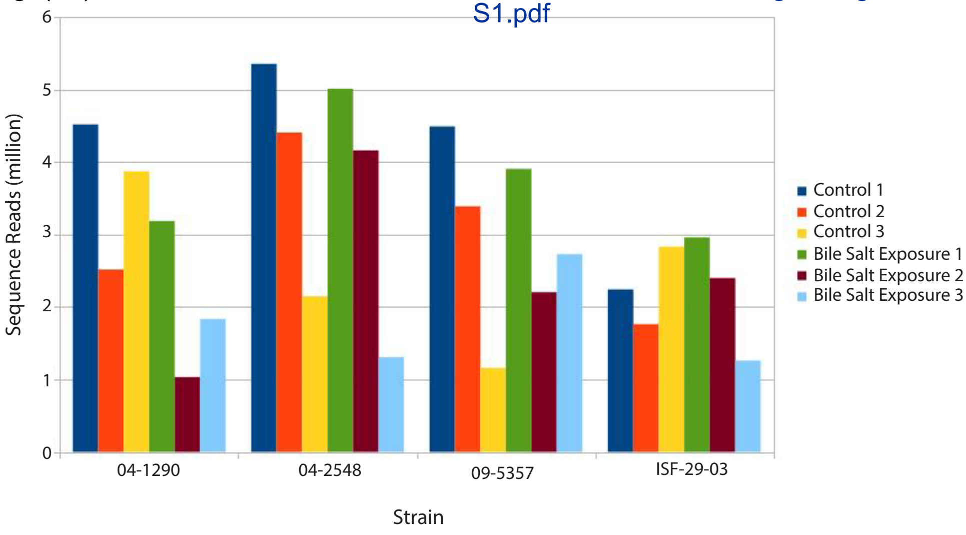

Figure S1- Total yield of RNA-Seq reads per sample.

 Click here to download Supplementary Material Files Bioinformatics Pipeline.docx

Supplementary Materials:

The following pipeline was used to process and analyze the RNA-seq sequence read data. Here we provide a sample pipeline with the understanding that the procedure was repeated for each of the four genomes used in our study (GENOME_NAME), and certain commands were repeated for both the test and control samples (CONTROL).

Bioinformatics Pipeline:

The reference genome was downloaded in fasta format for each assembly from NCBI.

Prokka was used to create an annotation from the fasta file, using the command:

>prokka --outdir GENOME_NAME –prefix GENOME NAME GENOME.fasta

Then the gffread package was used to convert the *.gff prokka output into an acceptable *.gtf file, using the command:

>gffread GENOME_NAME.gff -T -o *GENOME_NAME.gtf

The transcriptome data was downloaded from the MiSeq via BaseSpace in *.fastq format. To verify the quality of the data a check was preformed using the fastqc program, using the command:

>fastqc *.fastq.gz

A bowtie index for the sequence reads was constructed, using the commands:

>bowtie2-build GENOME.fasta btw/GENOME

Tophat was used to align the RNA-seq sequence reads to their respective genomes, using the command:

>tophat2 -p 8 -r 60 --library-type fr-firststrand -o GENOME_NAME_control -G GENOME_NAME.gtf /btw/GENOME GENOME_NAME_S$_L001_R1_001.fastq GENOME_NAME_S$_L001_R2_001.fastq

The triplicates of the test and control conditions were each merged, using the command:

>java -Xmx2g -jar /picard-tools-1.124/picard.jar MergeSamFiles OUTPUT=ALL.accepted_hits.bam INPUT= GENOME _NAME_ CONTROL/accepted_hits.bam

The cufflinks software package was used to identify differentially expressed genes, using the commands:

```
>cufflinks -p 8 -o $GENOME_NAME_CONTROL --library-type fr-firststrand --GTF
GENOME_NAME.gtf --frag-len-mean 262 --frag-len-std-dev 80 --no-update-check
GENOME_NAME_CONTROL/accepted_hits.bam
```

```
>ls -1 */transcripts.gtf >assembly_GTF_list.txt
```

```
>cuffmerge -p 8 -o merged -g GENOME_NAME.gtf assembly_GTF_list.txt
```

```
>cuffquant -p 8 --library-type fr-firststrand --frag-len-mean 262 --frag-len-std-dev 80 -o
$GENOME_NAME /merged.gtf GENOME_NAME_CONTROL/accepted_hits.bam
```

```
>cuffdiff -p 8 -L $GENOME_NAME_CONTROL, GENOME_NAME_TEST -o cuffdiff --library-type fr-
firststrand --frag-len-mean 262 --frag-len-std-dev 80 --no-update-check merged.gtf
GENOME_NAME_CONTROL/abundances.cxb GENOME_NAME_TEST/abundances.cxb
```

The statistically significant differentially expressed genes were selected from the output using the following custom script:

```
> grep -P "OK|gene_id" gene_exp.diff | sort -k 12n,12n | cut -f 3,5,6,8,9,10,12,13,14 | perl -ne
'@data=split("\t", $_); if ($data[6]<=0.05){print;}' > DE_genes.txt
```
